# Supplementary material for: Rapid turnover of life-cycle-related genes in the brown algae
Source: Genome Biol. 2019 Feb 14;20:35. doi: 10.1186/s13059-019-1630-6 (PMC6374913; doi:10.1186/s13059-019-1630-6)
Supplement: Supplementary file 3 — Protein alignments, corresponding Gblocks html file and CDS sequences for the lineage conserved gametophyte-biased genes. (ZIP 2109 kb) [file 13059_2019_1630_MOESM3_ESM.zip › Con_GA_alignments/Ec-28_003690.aa_ali.fasta-gb.htm]

Ec-28\_003690.aa.fasta.trans.aa\_ali.fasta


## Gblocks 0.91b Results

Processed file: **Ec-28\_003690.aa.fasta.trans.aa\_ali.fasta**  
Number of sequences: **2**  
Alignment assumed to be: **Protein**  
New number of positions: **957** (selected positions are underlined in blue)

```
                         10        20        30        40        50        60
                 =========+=========+=========+=========+=========+=========+
Ec-28_003690     MTSWGLELLSKLAESTSLDALREDFPRKSDEGLNPAQFLAVVRRHVEHGDFGFTAMNGGG
MSTRG.21718.1    --SW---------DDFSAYVLQLELLHDRKGAVENEHCFRHFATHLG--------IDG--
                                                                             


                         70        80        90       100       110       120
                 =========+=========+=========+=========+=========+=========+
Ec-28_003690     AGGGYPHGHGYNDVYGNASKVGEVFKEMDIRGDGEVCWEDFSAVSRVEYLPAPVEKLGLV
MSTRG.21718.1    ----------------------------HVRGGG---------ISRMDFLPSPVEKLGLV
                                                             ################


                        130       140       150       160       170       180
                 =========+=========+=========+=========+=========+=========+
Ec-28_003690     GKGNEVVHLIGQGPLEDDDKAPFQVVSGGGSDLARVSCSLRHDTAYKPHEVNAVHSIPGQ
MSTRG.21718.1    AKGNEVVHLVGQGPLEDDDEVPFRVVSGGGSDLARTSCSLRHDTAYKPHEVNAVHSLPGQ
                 ############################################################


                        190       200       210       220       230       240
                 =========+=========+=========+=========+=========+=========+
Ec-28_003690     DVVITSSSISSTGPHVLTVWDLPTQEERSTEKQFLPEMKSREETPTRQTVILWAPAVEMV
MSTRG.21718.1    DVVITSSSLGPSGPHVLTVWDLPTEEERSSEKQFLPALTCREETPTRQTVIFWAPAVEMV
                 ############################################################


                        250       260       270       280       290       300
                 =========+=========+=========+=========+=========+=========+
Ec-28_003690     LTGSSSNGSLLGWELGPLRQSAKLWLHDSGITHIHELVGHTGGAPHLVTASLDGTIAVCG
MSTRG.21718.1    LTGSGGNGCLLGWELGPLRQSAKLWLHDAGITHIHELVGHTGGDPHLVTASLDGTIAVCG
                 ############################################################


                        310       320       330       340       350       360
                 =========+=========+=========+=========+=========+=========+
Ec-28_003690     GAAKGGGRGDGVLQRLQAHERGIASMEFCLHHSLALSAGVFVNAVDTTPDILVWGLDRDR
MSTRG.21718.1    GTSKAGGYGDGVLQRLQAHEKGVAHMAFCLQHSLALSAGVFVNTVETTPDVLVWRLDRDR
                 ############################################################


                        370       380       390       400       410       420
                 =========+=========+=========+=========+=========+=========+
Ec-28_003690     GLLENDVQSRLRGHEGQVVGIAFTDEDLEVITGGADGVFLVWQMPSLVVKQRFTRAKNSP
MSTRG.21718.1    GLLEHDAQTRLRGHEGQVVGIAFAEEELEVLTGGADGTFLVWQIPSLVLKQRFASSKNSP
                 ############################################################


                        430       440       450       460       470       480
                 =========+=========+=========+=========+=========+=========+
Ec-28_003690     LVQQSVSCFALVPRSTDRPMLLVAGMKETAGLDIFARMEKRVHEELIKAEFCPYLQRLVT
MSTRG.21718.1    LVDQSVSCFSLVPRAVDRPLLLVAGMYKTTGLEIFARVEKRVHEALIKAEFCPYLQRFVT
                 ############################################################


                        490       500       510       520       530       540
                 =========+=========+=========+=========+=========+=========+
Ec-28_003690     VSARRVTIWDAKSGEALTTLTSERLLGNDQADITAFSSDHQGHKLVVGADTGEIRACFSH
MSTRG.21718.1    VSARRATVWDGKSGEALTTLTSERLLGSDQADITAFSLDHQGHKLVVGADTGRIRSCFSH
                 ############################################################


                        550       560       570       580       590       600
                 =========+=========+=========+=========+=========+=========+
Ec-28_003690     SGSVIRQLDPHNGPVNWLSFASRKTDMCVFSIGGDGELHVLDAADEKGYIKPPPPSAGRR
MSTRG.21718.1    SGSVIRQLDPHNGAVNWLAFASRKTDKCVFSVGGDGALHVLDDADAIGYVKPVSMADGRR
                 ############################################################


                        610       620       630       640       650       660
                 =========+=========+=========+=========+=========+=========+
Ec-28_003690     RKRLKYSLAARGDTISRDNAINHAEDDHDAIGSSDDDLSPGQ-LRSPRGASRLGRQSNFQ
MSTRG.21718.1    K--------------GTRNFSSHMSDGVGKGGSGKDDKADKKPCREGERRSSVGR-----
                                   ###################       ###########     


                        670       680       690       700       710       720
                 =========+=========+=========+=========+=========+=========+
Ec-28_003690     PKWGGSSEETGMATGGLTGNPASLGMGDCGAPRAAEVGACAGAEVGKATRKGGRSVLLRQ
MSTRG.21718.1    ------SVPVPDGHGSQTRGSATTDAAAEETAAVVDHGRGFEAEGGKRNRDKGWSVLLRQ
                       ################               #######################


                        730       740       750       760       770       780
                 =========+=========+=========+=========+=========+=========+
Ec-28_003690     VVFASNDSEDPGDVRHRSIDEDKNADAARPDRREVKSPSQQEEKQLLGNAEESSRESGML
MSTRG.21718.1    IAFASGDSDDEG----HDIGQGKSGGSLQPQSRGTWETSNGEP---LGSDDHTS------
                 ############      ########################                  


                        790       800       810       820       830       840
                 =========+=========+=========+=========+=========+=========+
Ec-28_003690     DHKGCPAEAPPASTGPAASSMKSRLASAARTLMSSAVRDELLEREILNGDKVNENGVRG-
MSTRG.21718.1    -----AMDDGSDSTAAVGSPSKPGFARAARALMSSSLREELLEKELRSEEKDGISKAGGK
                             ############################################### 


                        850       860       870       880       890       900
                 =========+=========+=========+=========+=========+=========+
Ec-28_003690     DLIIAKPTFDMTACAADDHLSIIATAVTSDGGVEATMPAESGGAPNKDQMASTRVGRASS
MSTRG.21718.1    GLLLAKPRFDMTACAADDHLSMIATAATSDGGIEAATPAKSGGNADHNSTTSTRVGRPSS
                  ###########################################################


                        910       920       930       940       950       960
                 =========+=========+=========+=========+=========+=========+
Ec-28_003690     SLHLWDAERMSLLGTLLAPCLALTRDLTDAAAAFSVHPHCAAVAQPPVDPSATDPP----
MSTRG.21718.1    SLHLWDAERLTLLGTLLLPCLALTRDLANAAAAFGIQTACVPGATRAVDRTESDPPPCVN
                 ########################################################    


                        970       980       990      1000      1010      1020
                 =========+=========+=========+=========+=========+=========+
Ec-28_003690     ------STPVESLHGNNKDMDGENPTATVGNET-------T---TTTSQPPVRHAAAATD
MSTRG.21718.1    TKDLTKGSAREKGHDHDKACQDE-PTAASDTESGRPAAESTPSRVCDIEPPPSGNVVAQM
                           #############                          ###########


                       1030      1040      1050      1060      1070      1080
                 =========+=========+=========+=========+=========+=========+
Ec-28_003690     KQHAATTKTGTGLNRPALVTALMFLSPYPLLMGASTGGAVVVWRTLDCVCVQGQMPAVQV
MSTRG.21718.1    RIPGAAVRAGTGLNRPALVNSLMFLSPYPLLVGASTGGAVVLWRASDCVCV---------
                 ###################################################         


                       1090      1100      1110      1120      1130      1140
                 =========+=========+=========+=========+=========+=========+
Ec-28_003690     DNKKYGRSPFSFNAQAILLPADLASLRKKNISAAGGSGGH---LREESGAKEEE-TLTCL
MSTRG.21718.1    --------------QAILLPADLASLRKERRGSGDDCNGHHDGMLEEAGSRETEGHITCL
                               ##############                                


                       1150      1160      1170      1180      1190      1200
                 =========+=========+=========+=========+=========+=========+
Ec-28_003690     ARGTFTGSTGEETTSIYVGNGNGDIVAAHLSPTELAELSGDAKGPVQCQRRPNHNPYRAV
MSTRG.21718.1    ARGTCT-SVGGEVTVLYAGNGSGDVVVAHLSPNELAELSGDAGGPVPCQRRDNHNPYRAV
                        #####################################################


                       1210      1220      1230      1240      1250      1260
                 =========+=========+=========+=========+=========+=========+
Ec-28_003690     RMELTPQVARFSLQAACSAQKQAAAGGNGEAGVAGGAHADMPRRRQKGSSSDGVGAYKFA
MSTRG.21718.1    RVKLTPQAARISFQAACRAQGRAVGRTQRTS-----------RNGPKRSPSDPRGGFNFA
                 #######################                   ##################


                       1270      1280      1290      1300      1310      1320
                 =========+=========+=========+=========+=========+=========+
Ec-28_003690     VPSSGFHSAWAAHSGAVTSISWIRSPPSLLSSSADGLAKIWKPDGSAMLGQLDINNRRPE
MSTRG.21718.1    VPSSQFQAAWAX------------------------------------------------
                 ###########                                                 


                       1330      1340      1350      1360      1370      1380
                 =========+=========+=========+=========+=========+=========+
Ec-28_003690     RGMYLAAEASRHWAFVPGSGGPADCRHEEDGLDQEKLTHDTAKATTATTDSVVKKTIGNS
MSTRG.21718.1    ------------------------------------------------------------
                                                                             


                       1390      1400      1410      1420      1430      1440
                 =========+=========+=========+=========+=========+=========+
Ec-28_003690     GELTVNDTSSSTKTNSFRGGGGNVEGDLHDDVGDEADSTGAGPGRNERRGRSGRQQQQQH
MSTRG.21718.1    ------------------------------------------------------------
                                                                             


                       1450      1460      1470      1480      1490      1500
                 =========+=========+=========+=========+=========+=========+
Ec-28_003690     DREKQRDRNEKTQPDEGGLRSPDPTRQLQDFEEALEASSSKRTPKAERFHLGRPMSLATM
MSTRG.21718.1    ------------------------------------------------------------
                                                                             


                       1510      1520      1530      1540      1550      1560
                 =========+=========+=========+=========+=========+=========+
Ec-28_003690     QPIRKMPLCGKRWRTKSAPWPAEDESLLERSVSEQGLPTAGSVRVGTHKGMRGYVRPTAG
MSTRG.21718.1    ------------------------------------------------------------
                                                                             


                       1570      1580      1590      1600      1610      1620
                 =========+=========+=========+=========+=========+=========+
Ec-28_003690     SAEDAALKRTKSRGATGVVLKDTMGEESGRNSLQGRPLGEWVAQPEGENAVPCIDGTGEP
MSTRG.21718.1    ------------------------------------------------------------
                                                                             


                       1630      1640      1650      1660      1670      1680
                 =========+=========+=========+=========+=========+=========+
Ec-28_003690     ANTVARGAGESPMDKTIKGLPPVAVLAGSHGGRLIASGITIAGDRGGGGFNVKAARAAAR
MSTRG.21718.1    ------------------------------------------------------------
                                                                             


                       1690      1700      1710      1720      1730      1740
                 =========+=========+=========+=========+=========+=========+
Ec-28_003690     TATRRAALAEELAKEGECNRRKRNKADRRARTTIGAGASRQQRQGTQHGRELHGGRGPSS
MSTRG.21718.1    ------------------------------------------------------------
                                                                             


                       1750      1760      1770      1780      1790      1800
                 =========+=========+=========+=========+=========+=========+
Ec-28_003690     WGLEQGGNRKSSRRAISGNGGDAIGAAGEISPGGVPHNAGGSLAPSFGGMAQSVGSVGSG
MSTRG.21718.1    ------------------------------------------------------------
                                                                             


                       1810      1820      1830      1840      1850      1860
                 =========+=========+=========+=========+=========+=========+
Ec-28_003690     GGGSIVRTEASESTVVLPAEGAADTRKLRELNEDSVVWASTRRGSGRSSSSSKNRRQRHD
MSTRG.21718.1    ------------------------------------------------------------
                                                                             


                       1870      1880
                 =========+=========+
Ec-28_003690     KEEGSGQVETIRRLVHMGLF
MSTRG.21718.1    --------------------
```

```
Parameters used
Minimum Number Of Sequences For A Conserved Position: 2
Minimum Number Of Sequences For A Flanking Position: 2
Maximum Number Of Contiguous Nonconserved Positions: 8
Minimum Length Of A Block: 10
Allowed Gap Positions: None
Use Similarity Matrices: Yes
```

```
Flank positions of the 13 selected block(s)
Flanks: [105  600]  [619  637]  [645  655]  [667  682]  [698  732]  [739  762]  [793  839]  [842  956]  [971  983]  [1010  1071]  [1095  1108]  [1148  1223]  [1243  1271]  

New number of positions in Ec-28_003690.aa.fasta.trans.aa_ali.fasta-gb:  957  (50% of the original 1880 positions)
```
